# Case Report: Late simultaneous metastasis of renal clear cell carcinoma to the ampulla and breast

**DOI:** 10.3389/fonc.2025.1604170

**Published:** 2025-08-20

**Authors:** Guofeng Dai, Shuya Li, Lei Sun, Gangpu Wang

**Affiliations:** ^1^ Department of Thyroid and Breast Surgery, The Fourth People’s Hospital of Jinan, Jinan, China; ^2^ Department of Critical Care Medicine, The Fourth People’s Hospital of Jinan, Jinan, China; ^3^ Department of Pathology, Shandong Public Health Clinical Center, Jinan, China

**Keywords:** renal cell carcinoma, ampullary mass, obstructive jaundice, metastatic breast cancer, cancer

## Abstract

We present the case of a 67-year-old female who developed simultaneous metastases to the ampulla of Vater and the breast. Her medical history is significant for a radical nephrectomy performed twenty-one years prior for renal cell carcinoma (RCC). The patient was referred for evaluation due to the development of progressive jaundice, fatigue, and weight loss. A computed tomography (CT) scan of the abdomen revealed a 40 mm nodule located at the ampulla of Vater, accompanied by dilatation of the bile duct, indicative of obstructive jaundice. Additionally, ultrasonography identified a mass approximately 20 mm in size in the lateral portion of the left breast. The patient underwent a pancreatoduodenectomy to address the ampullary mass and a lumpectomy for the breast lesion. Histological examination of the resected specimens confirmed the diagnosis of metastases from RCC in both the ampulla of Vater and the breast.

## Introduction

RCC is the third most common type of all primary renal tumors, after prostate and bladder cancer _(_ 1_)._ Clear cell renal cell carcinoma is the most common subtype of renal epithelial tumors, accounting for approximately 2% of all malignant tumors (2). It originates from renal tubular epithelial cells (3). Compared to other subtypes, Clear cell renal cell carcinoma typically exhibits a higher degree of malignancy, a broader mutational spectrum, a higher rate of metastasis and recurrence, and a poorer clinical prognosis (4). Approximately one-third of patients with RCC either present with metastatic disease at the time of diagnosis or develop metastases within months or years following initial treatment (3). The time to metastasis generally occurs years beyond primary cancer presentation. Approximately one-third of patients with RCC either present with metastatic disease at the time of diagnosis or develop metastases within months or years following initial treatment. Two-thirds of RCC are localized, which has a 5-year survival rate of over 90%. This survival rate, however, drops to 14% when the disease is metastatic (5). RCC metastasis to the ampulla and breast remains a rarity and simultaneous metastases of RCC to the ampulla and breast is even rarer. To the best of our knowledge, this is the first report of clear cell RCC simultaneous metastasized to the ampulla of Vater and breast.

The treatment of metastatic clear cell renal carcinoma mainly includes surgical intervention, postoperative adjuvant targeted therapy, and immunotherapy. While surgical resection remains the primary treatment modality for localized RCC, the persistent recurrence rate highlights a significant unmet need for effective adjuvant therapies. In recent years, advancements in immunotherapy and targeted therapies have revolutionized the treatment landscape of RCC (6). Research into the biology of renal cell carcinoma has led to the development of novel targeted therapies, including mTOR (mammalian target of rapamycin) inhibitors like Temsirolimus, as well as inhibitors of tyrosine kinase receptors from the division kinase domain family, such as Sunitinib and Sorafenib. These therapies block tumor angiogenesis by inhibiting growth factors through the vascular endothelium. Kidney cancer has a unique immune microenvironment in the tumor CD8+T cells are extensively infiltrated, but their toxic function is dysfunctional and they are not used for killing which makes the application of immune checkpoint inhibitor (ICI) in renal cancer attract wide attention (7). Immunotherapy can prolong progression-free survival in patients while enhancing the efficacy of anti-vascular targeted therapies. The synergistic effect of the two makes immunotherapy a first-line treatment option for patients with advanced renal cancer.

## Case report

**Figure 1:**
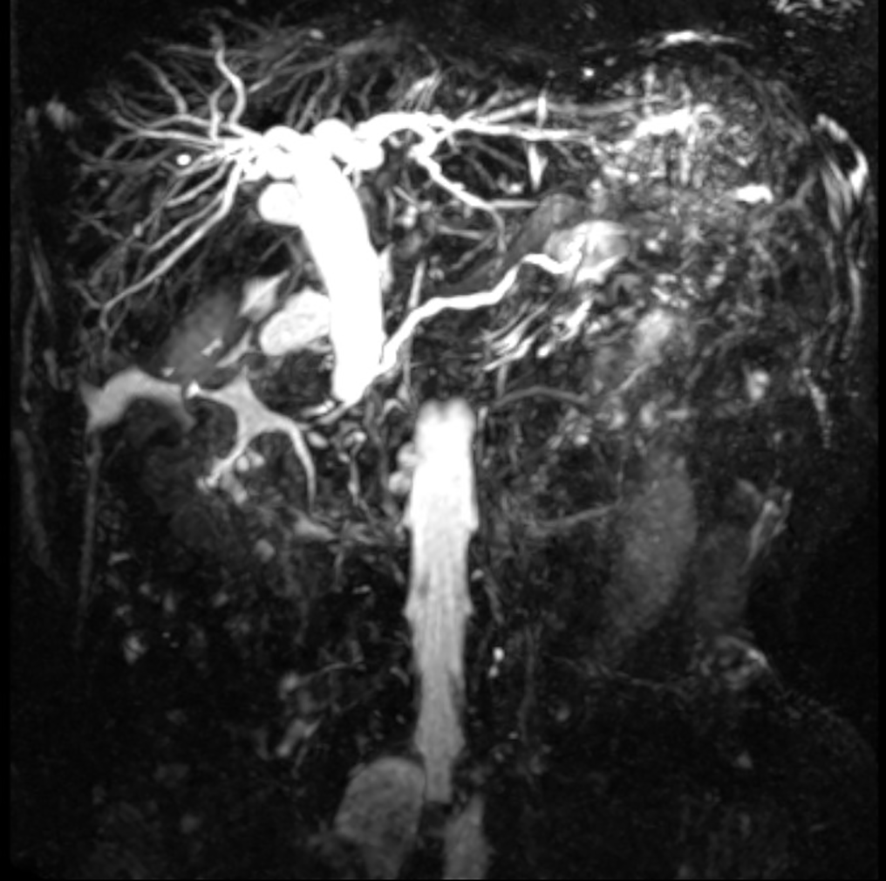
MRI showing dilatation of bile.

**Figure 2:**
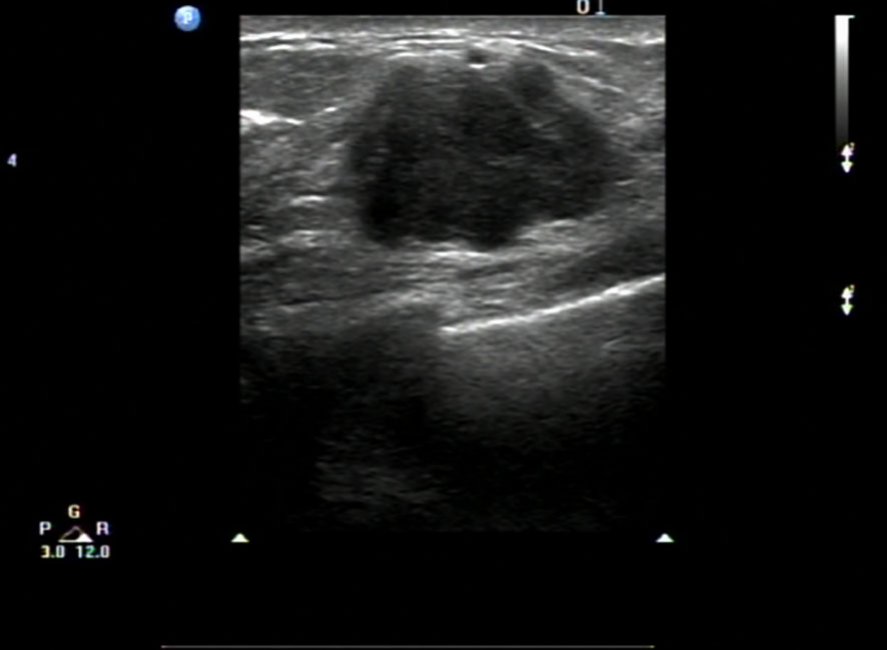
US performed shows a well-circumscribed, hypoechoic mass measuring 20 mm.

A 67-year-old female patient presented with a 4-week history of fatigue, anorexia, jaundice, nausea, and vomiting. She also experienced intermittent diarrhea and had lost 4 kilograms in recent months. She had a history of radical excision of the left kidney due to RCC in 1997. Histological examination revealed clear cell carcinoma staged as T2aN0, and she received no further adjuvant treatment. Results of pertinent laboratory studies on admission showed the following: serum glutamic-pyruvic transaminase (GPT, ALT) at 208.4 U/L, glutamic-oxaloacetic transaminase (GOT, AST) at 143.4 U/L, total bilirubin (TBIL) at 124 μmol/L, direct bilirubin (DBIL) at 57.8 μmol/L, and carcinoembryonic antigen (CEA) at 2.31 ng/mL. Magnetic resonance imaging (MRI) revealed dilatation of the bile duct (**Figure 1**). An abdominal computed tomography (CT) scan confirmed the presence of an irregularly shaped tumor in the ampullary region, with indistinct borders with the pancreas, and no evidence of regional lymph node metastasis or thrombus within the inferior vena cava or renal veins. On clinical examination, we found a mass of approximately 20mm mass in the lateral portion of the left breast. Breast ultrasound revealed a well-circumscribed, hypoechoic mass measuring 20 mm at the same site. (**Figure 2**) Fine needle aspiration (FNA) was taken from breast mass, but it did not capture breast duct epithelial cells.

**Figure 3:**
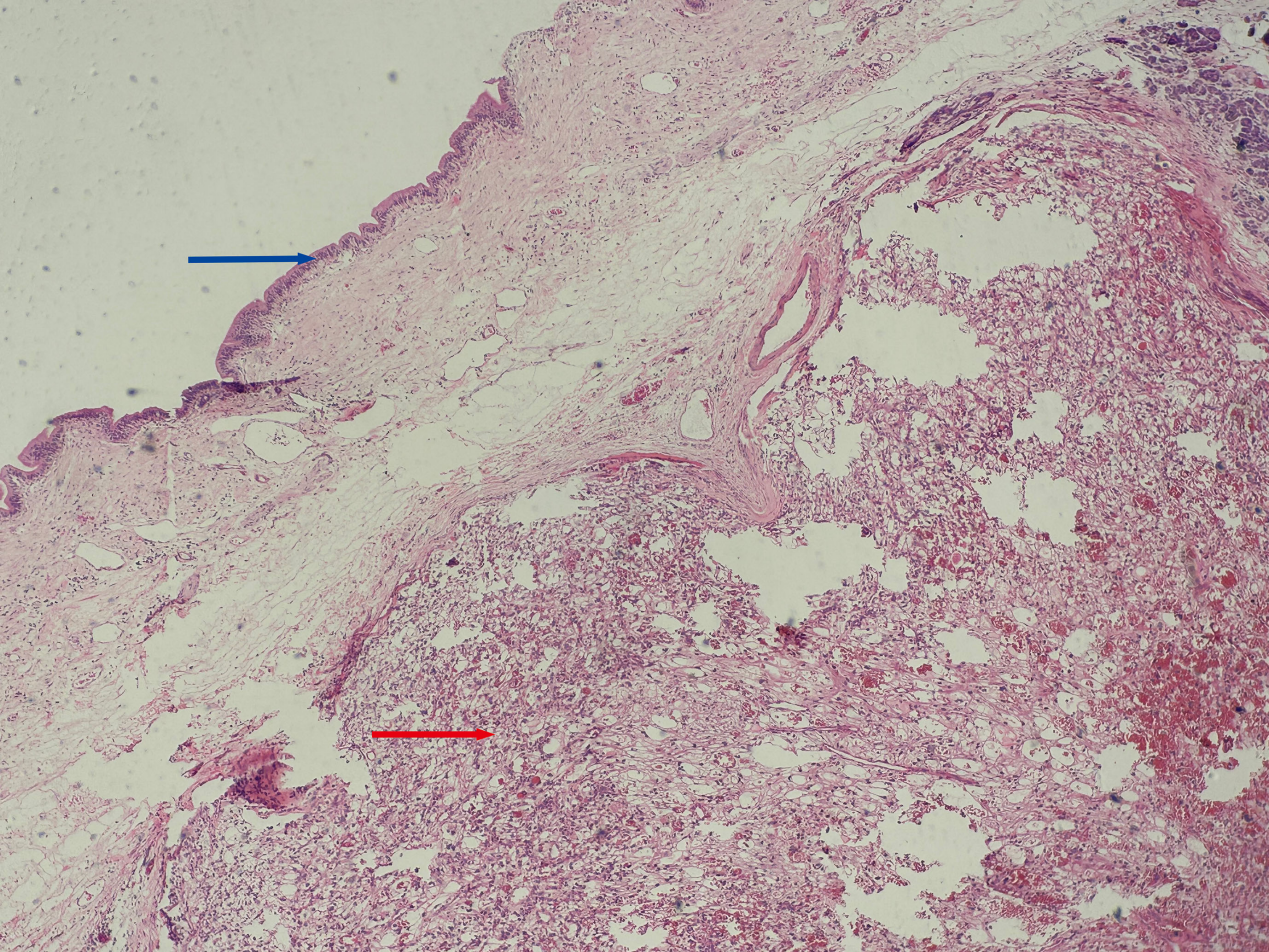
Histopathological view of the ampullary tumor. (Hematoxylin and Eosin stain, original magnification×100) Blue arrow: Shows the mucous epithelium of the ampulla. Red arrow: Shows clear cell renal cell carcinoma with solid, nested growth, invading the ampullary wall.

**Figure 4:**
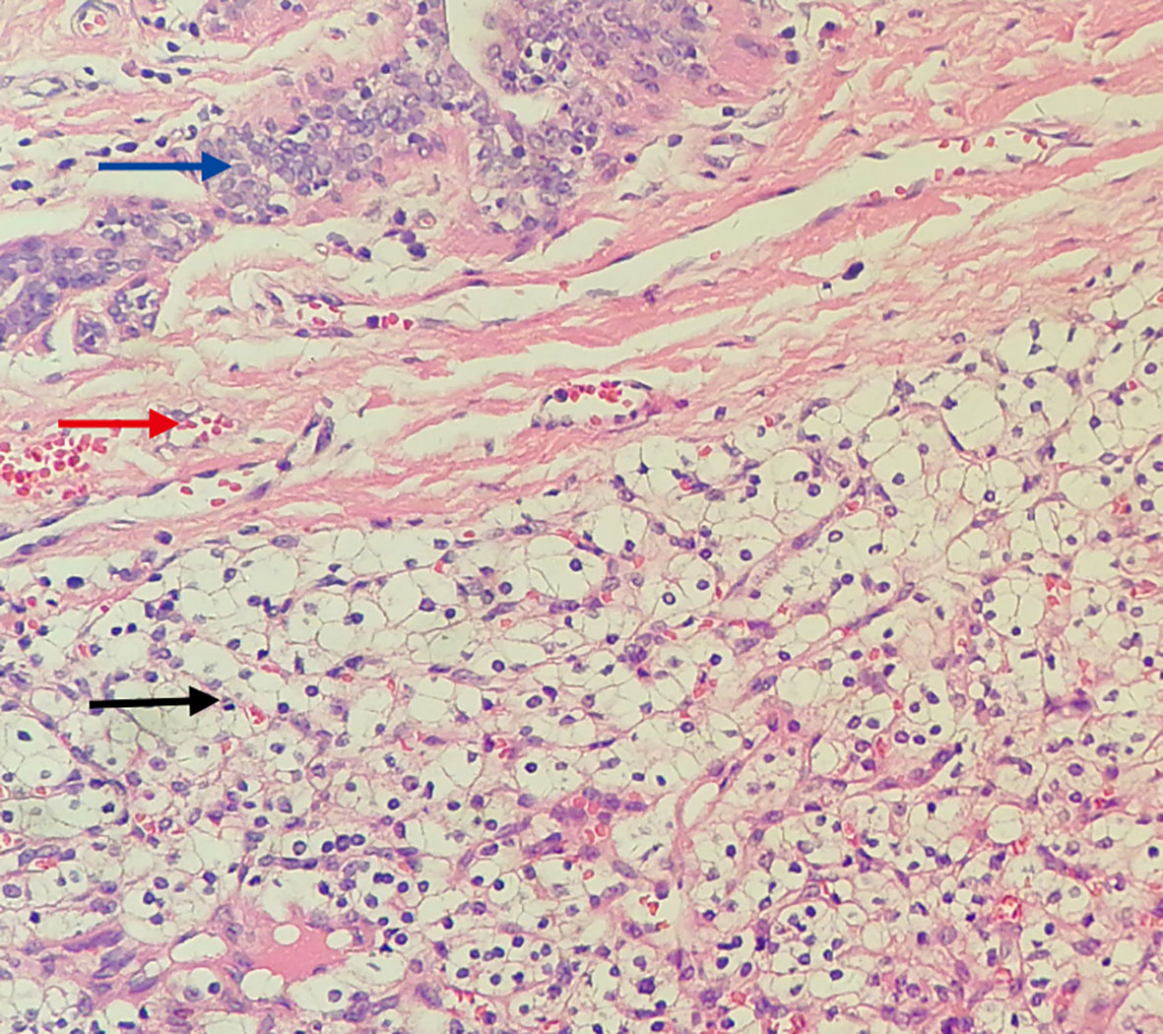
Histopathological view of the breast tumor. (Hematoxylin and Eosin stain, original magnification×100) Blue arrow: Shows the mammary duct. Red arrow: Shows fibrous tissue hyperplasia forming a pseudocapsule. Black arrow: Shows metastatic clear cell renal cell carcinoma tissue, with clear, transparent cytoplasm, shrunken nuclei, and a stroma densely populated with a capillary network.

**Figure 5:**
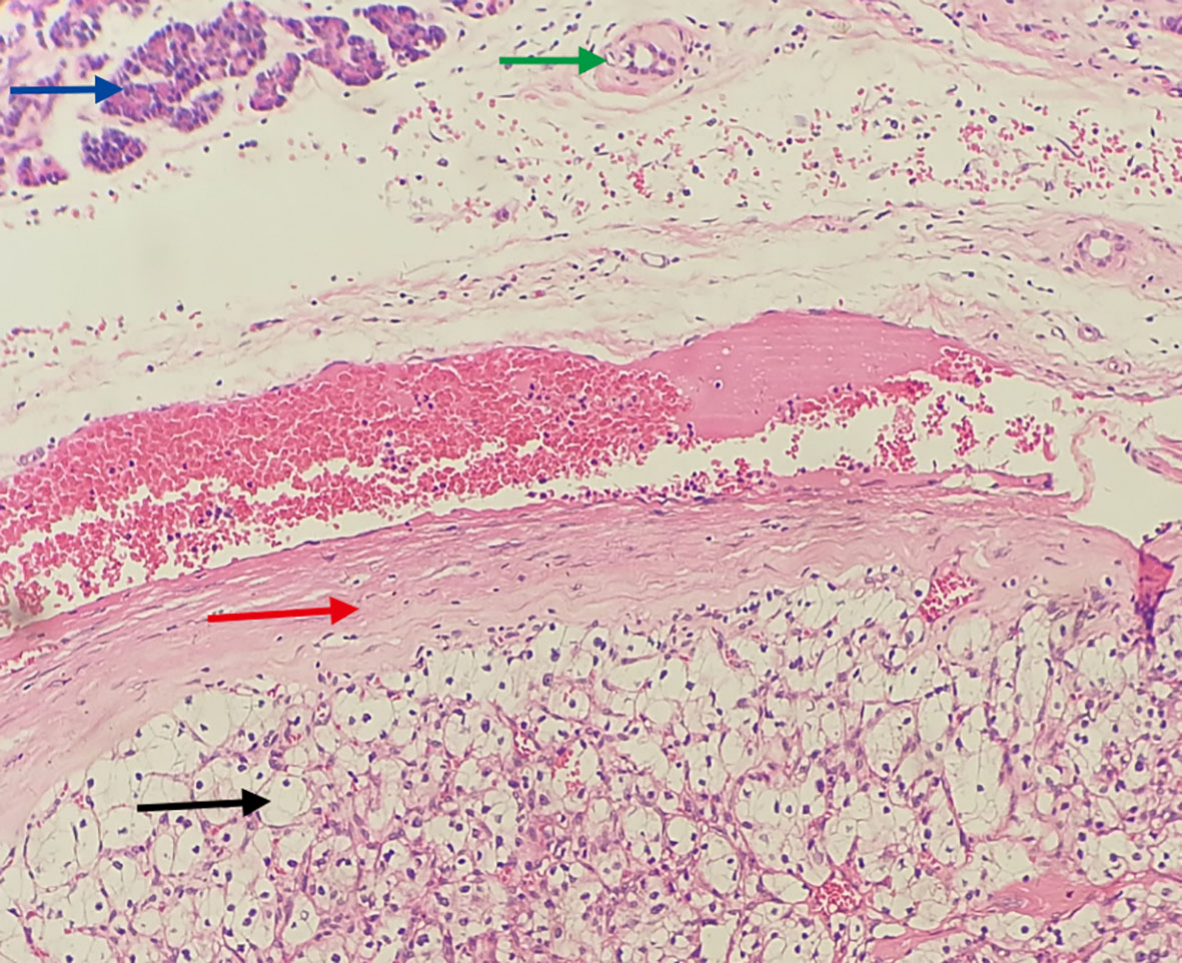
Translates to cancerous tissue invading the pancreas, with indistinct borders. (Hematoxylin and Eosin stain, original magnification×100) Blue arrow: Shows pancreatic acini (cytoplasm eosinophilic, nuclei mild, arranged in an acinar pattern). Green arrow: Indicates the pancreatic duct. Red arrow: Shows fibrous tissue hyperplasia forming a pseudocapsule. Black arrow: Shows metastatic clear cell renal cell carcinoma tissue, with clear, transparent cytoplasm, shrunken nuclei, and a stroma densely populated with a capillary network.

**Figure 6:**
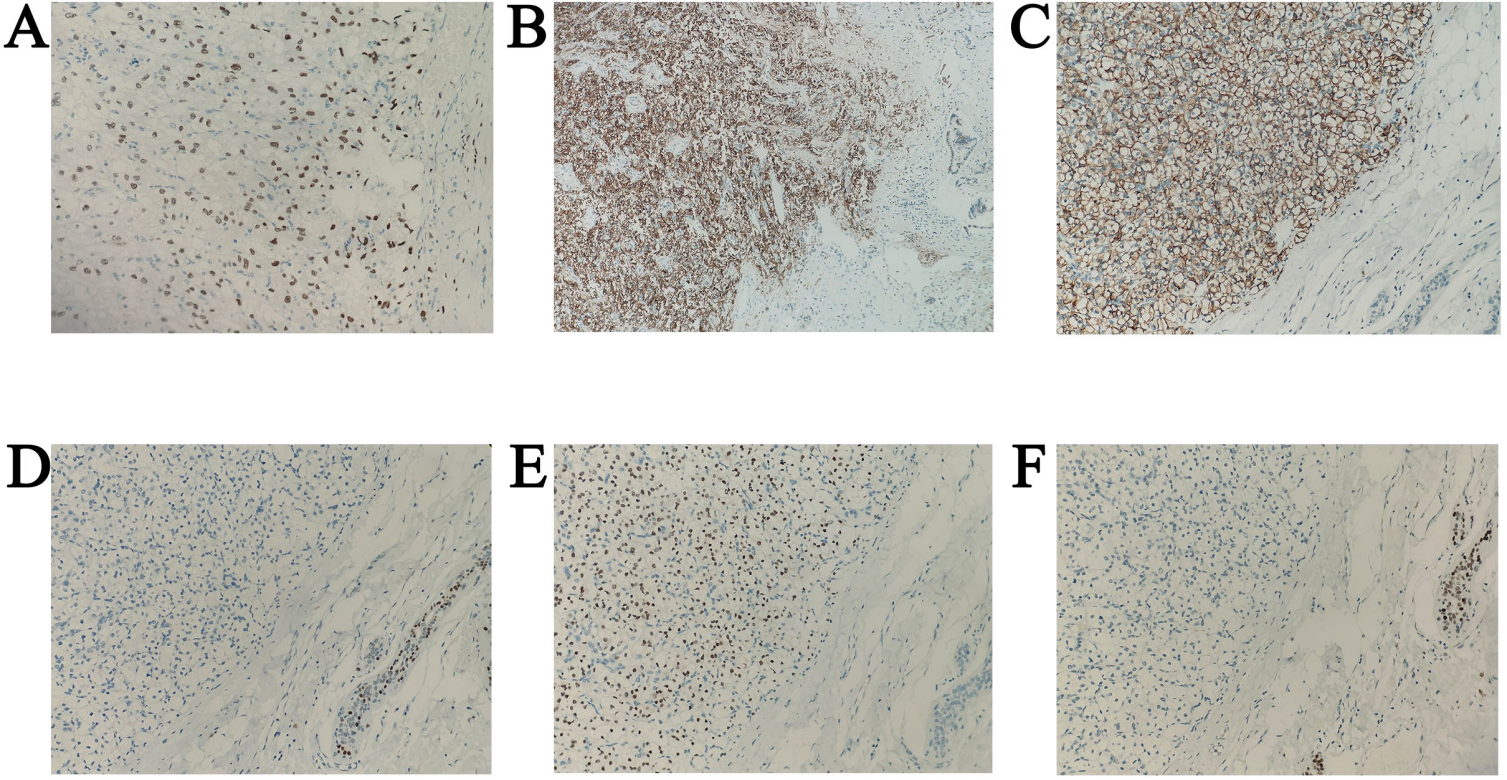
Immunohistochemical image. **(A)** CA9 positivity in metastatic breast tumor, original magnification ×200. **(B)** A9 positivity in metastatic tumors of the ampulla of Vater, original magnification ×100. **(C)** PAX8 positivity in metastatic breast tumor, original magnification ×200. **(D)** PAX8 positivity in metastatic tumors of the ampulla of Vater, original magnification ×200. **(E)** ATA3 negativity in metastatic breast tumor, original magnification ×200. **(F)** TRPS1 negativity in metastatic breast tumor, original magnification ×200.

Based on the preoperative diagnostic assessments, a provisional diagnosis of synchronous malignancies involving the ampulla of Vater and the breast has been established. The potential for renal carcinoma metastasis remains a consideration within the differential diagnosis. It is advised that the patient undergo a histological puncture biopsy of the left breast mass prior to surgery, as well as endoscopic retrograde cholangiopancreatography (ERCP). Post-surgery, targeted therapy and immunotherapy are recommended. However, the patient’s family strongly insists on open surgical treatment. Total pancreaticoduodenectomy and lumpectomy were performed in August 2018. Pathological examination resulted in the diagnosis of the lesions as metastases to the ampulla of Vater and breast from renal cell carcinoma (RCC) of the clear-cell type. (**Figures 3**, **4**). Postoperative pathology revealed the tumor had invaded the ampullary wall and demonstrated an indistinct boundary with the pancreatic tissue. (**Figure 5**). The immunohistochemical positivity for various markers, such as vimentin, CD-10, and epithelial membrane antigen (EMA), supports the diagnosis. All lymph nodes and the margins of resection from the common bile duct, pancreas, duodenum, and jejunum were negative for tumor. We examined the surgical specimens, supplementary immunohistochemical staining was performed, and a retrospective review was conducted. The results are as follows (**Figure 6**). The postoperative course was uneventful, and there were no significant complications during the hospital stay. Our patient was discharged 20 days after surgery. The postoperative course was smooth, and the patient was discharged. Due to economic constraints, they refused targeted therapy and immunotherapy. After closely following up for 8 months post-discharge, the patient passed away due to cachexia and respiratory and circulatory failure.

## Discussion

The cancer cells of RCC are renowned for their exceptional adaptability to the microenvironment, which confers an elevated propensity for metastasis and unforeseen dissemination to various organs and tissues. Hematogenous spread is the predominant pathway for metastatic dissemination in RCC (8). The most frequent sites for RCC metastasis are the lung parenchyma, accounting for 45.2%, followed by the skeleton at 29.5%, lymph nodes at 20.8%, liver at 20.3%, adrenals at 8.9%, and brain at 8.1% (9). Metastasis to the breast and periampullary region is infrequent. A review of the English literature reveals that periampullary metastasis of RCC is exceptionally rare, with only 19 documented cases reported worldwide to date (10). A review of the English literature indicates a greater number of studies on the metastasis of renal clear cell carcinoma to the breast compared to the ampulla; however, none provide specific numerical data.

The occurrence of breast metastases from extramammary tumors is relatively uncommon, with reported incidences in clinical literature ranging from 0.5% to 2% (11). Among primary extramammary malignancies, the incidence of renal cancer ranks fifth, following lymphoma, melanoma, lung cancer, and ovarian cancer (12). The metastatic pathway of tumor cells typically involves the right ventricle of the heart, followed by transit through the inferior vena cava and entry into the pulmonary circulation, ultimately reaching the breast. Additionally, cancer cell transport can also occur via the paravertebral venous plexus (13). The majority of primary breast malignancies present with spiculated lesions, microcalcifications, architectural distortion, and asymmetrical density (14). Additionally, these lesions often demonstrate a relatively abundant blood supply (15). The tumor was surrounded by a fibrous tissue pseudocapsule, which was crucial in differentiating RCC during the differential diagnosis process (16). Immunohistochemical analysis showed strong positive staining for CD10, vimentin, and EMA, supporting the diagnosis of renal cell carcinoma RCC (17). Establishing an accurate diagnosis of metastatic breast tumors requires core needle biopsy, pathological histological analysis, and immunohistochemical analysis. These procedures are essential as they indicate the type and origin of the tumors, which is crucial for determining appropriate systemic or surgical treatment for metastatic breast cancer (18).

The prognosis for patients with metastatic RCC is indeed very poor, with median survival times typically ranging from 6 to 12 months. Furthermore, the two-year survival rate for these patients varies between 10% and 20%. The rate of achieving a complete response to nonsurgical treatment is less than 15%, while surgical intervention is associated with improved overall survival outcomes (19). For patients with localized disease, surgical intervention is the recommended treatment modality. In advanced stages, a combination of tyrosine kinase inhibitors and PD-1/PD-L1 inhibitors has demonstrated efficacy in enhancing response rates and prolonging progression-free survival (20). The performance of mastectomy and lymph node dissection is considered unwarranted, as they do not provide curative benefits.

RCC of the ampulla is extremely rare, with obstructive jaundice often being the primary clinical manifestation (21). Renal cell carcinoma is not the only malignancy that can metastasize to the ampulla of Vater; other cancers, including melanoma, breast carcinoma, squamous cell carcinoma of the larynx, and cervical carcinoma, have also been reported to disseminate to this anatomical site (22). The patient’s clinical manifestations may include nonspecific abdominal discomfort, jaundice, or upper gastrointestinal bleeding. Macroscopically, the specimen may appear as polypoid or irregularly shaped masses, with a very soft texture that is prone to fragmentation, and it may also present as superficial ulcers. When a patient with RCC presents with painless jaundice, especially when a CT scan does not reveal obstructive causes in the pancreas or elsewhere, the possibility of ampullary metastasis should be considered (23). Isolated ampullary metastases are primarily treated with pancreaticoduodenectomy, with a median survival time of 26 months post-surgery. Among secondary malignant tumors with ampullary metastasis, RCC has the best prognosis (24).

Metastatic renal clear cell carcinoma has a poor prognosis and is unresponsive to radiotherapy and chemotherapy. Targeting the VEGF and mTOR pathways is crucial in treatment, with targeted therapies including tyrosine kinase inhibitors (TKIs) such as sunitinib and mTOR inhibitors like temsirolimus (25). Additionally, this tumor has a unique immune microenvironment characterized by significant infiltration of CD8+ T cells, leading to the widespread use of immune checkpoint inhibitors (ICIs) in treatment. Treatment options for metastatic renal clear cell carcinoma are guided by the International Metastatic Renal Cell Carcinoma Database (IMDC) score. Low-risk patients are recommended to receive monotherapy with targeted agents, while high-risk patients are advised to undergo combination targeted immunotherapy or dual immunotherapy.

In the KEYNOTE-426 study, 861 patients with advanced clear cell renal cell carcinoma were randomly assigned. Of these, 432 patients received intravenous pembrolizumab combined with oral axitinib, while 429 patients received oral sunitinib as monotherapy. In the low-risk group, no statistically significant differences were observed in overall survival (OS) or progression-free survival (PFS) between the two combinations. This trial demonstrated that immunotherapy did not offer any additional benefits for the low-risk subgroup of patients (26).

The mTOR domain can be targeted by tisorolimus (CCI-779) through a complex formed with binding proteins. This interaction inhibits the mTOR signaling pathway, leading to the suppression of tumor cell proliferation, transformation, and angiogenesis (27). Studies have shown that first-line treatment with tisorolimus is beneficial for patients with high-risk metastatic renal cell carcinoma, as classified by the MSKCC, as well as for those with non-clear cell renal carcinoma. Conversely, in patients with renal cancer who experience progression after initial treatment with sunitinib, sorafenib—a VEGF pathway inhibitor—has demonstrated superiority over tisorolimus, an mTOR pathway inhibitor. The NCCN guidelines strongly recommend classifying tisorolimus as a Class I recommendation solely for patients at high risk of developing metastatic renal cancer, as determined by the MSKCC classification.

The immunogenicity of renal cell carcinoma is notably high, with approximately 30% of cases exhibiting overexpression of PD-L1 (28). The expression of PD-L1 in renal cell carcinoma cells can stimulate the production of antigenic mimics, resulting in heterogeneous tumor growth and potentially inducing apoptosis in circulating T cells (29). For patients with moderate to high-risk IMDC scores, treatment options include targeted immunotherapy or dual immunotherapy. Patients with IMDC risk stratified by CheckMate 214 who received the combination of nivolumab and ipilimumab demonstrated significantly improved overall survival (OS) and objective response rates compared to those treated with sunitinib (30). The efficacy of pembrolizumab in combination with axitinib was evaluated in KEYNOTE 426 across all IMDC risk stratifications, showing a significant improvement in progression-free survival (PFS), overall survival (OS), and overall response rate (ORR). This effect was particularly pronounced in moderate-to-high-risk mRCC patients (26). A meta-analysis of phase III randomized controlled trials (RCTs) compared the combination therapy of pembrolizumab plus a tyrosine kinase inhibitor (TKI) with sunitinib in treatment-naive advanced renal cell carcinoma (aRCC). The overall survival (OS) probabilities for patients treated with the pembrolizumab-TKI combination at 18, 24, and 36 months were 83%, 76%, and 63%, respectively. In comparison, patients treated with sunitinib had corresponding OS probabilities of 72%, 65%, and 54% at these time points. Regarding progression-free survival (PFS), the probabilities for patients receiving the pembrolizumab-TKI combination at 18, 24, and 36 months were 52%, 43%, and 24%, respectively, while those receiving sunitinib had PFS probabilities of 33%, 24%, and 15% (31).

## Conclusions

Renal cell carcinoma (RCC) can develop atypical metastases several years after diagnosis. While metastases to the breast and ampulla are rare in RCC, our case is unique as it presents with isolated simultaneous metastases in these locations. In this context, targeted therapy and immunotherapy play a crucial role in the first-line treatment of metastatic RCC.

## Data Availability

The raw data supporting the conclusions of this article will be made available by the authors, without undue reservation.
